# Extracellular vesicles: mediators of intercellular communication in tissue injury and disease

**DOI:** 10.1186/s12964-021-00787-y

**Published:** 2021-10-16

**Authors:** Greg Berumen Sánchez, Kaitlyn E. Bunn, Heather H. Pua, Marjan Rafat

**Affiliations:** 1 Department of Chemical and Biomolecular Engineering, Vanderbilt University, Nashville, TN USA; 2 Department of Pathology, Microbiology, and Immunology, Vanderbilt University Medical Center, Nashville, TN USA; 3 Department of Biomedical Engineering, Vanderbilt University, Nashville, TN USA; 4 Department of Radiation Oncology, Vanderbilt University Medical Center, Nashville, TN USA

**Keywords:** Extracellular vesicles, Immune response, Intercellular communication, Reprogramming, Tumor microenvironment, Metabolic disorders, Lung inflammation

## Abstract

Intercellular communication is a critical process that ensures cooperation between distinct cell types and maintains homeostasis. EVs, which were initially described as cellular debris and devoid of biological function, are now recognized as key components in cell–cell communication. EVs are known to carry multiple factors derived from their cell of origin, including cytokines and chemokines, active enzymes, metabolites, nucleic acids, and surface molecules, that can alter the behavior of recipient cells. Since the cargo of EVs reflects their parental cells, EVs from damaged and dysfunctional tissue environments offer an abundance of information toward elucidating the molecular mechanisms of various diseases and pathological conditions. In this review, we discuss the most recent findings regarding the role of EVs in the progression of cancer, metabolic disorders, and inflammatory lung diseases given the high prevalence of these conditions worldwide and the important role that intercellular communication between immune, parenchymal, and stromal cells plays in the development of these pathological states. We also consider the clinical applications of EVs, including the possibilities for their use as novel therapeutics.

**Video Abstract**

**Supplementary Information:**

The online version contains supplementary material available at 10.1186/s12964-021-00787-y.

**Figure:**
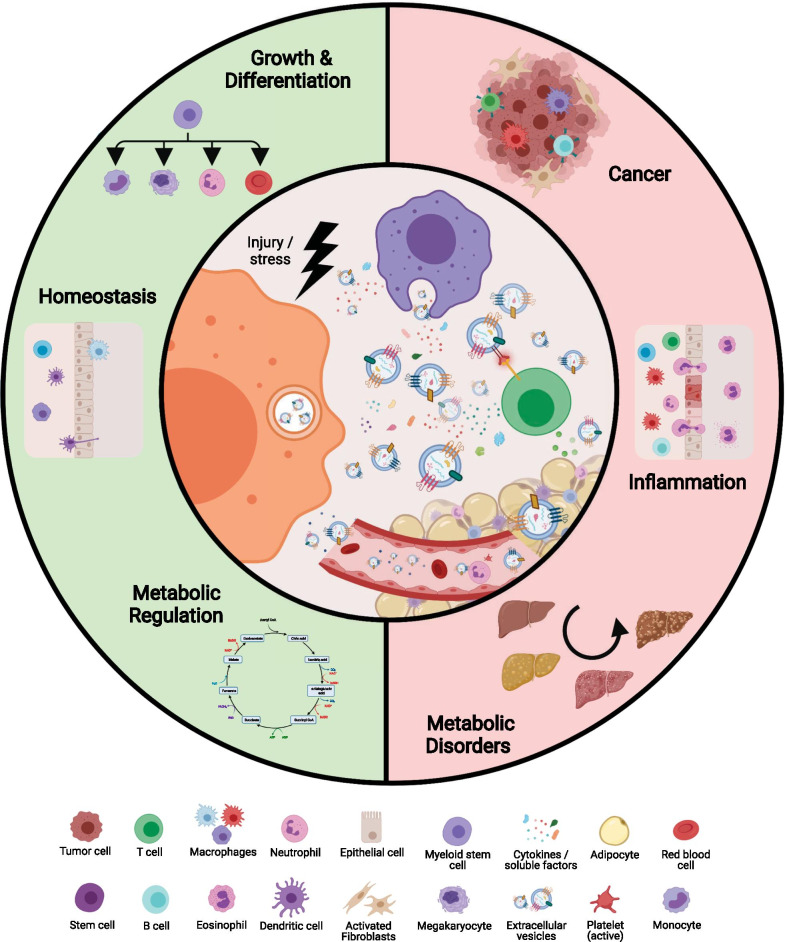
While intercellular communication through extracellular vesicles (EVs) is key for physiological processes and tissue homeostasis, injury and stress result in altered communication patterns in the tissue microenvironment. When left unchecked, EV-mediated interactions between stromal, immune, and parenchymal cells lead to the development of disease states

## Background

**Fig. 1:**
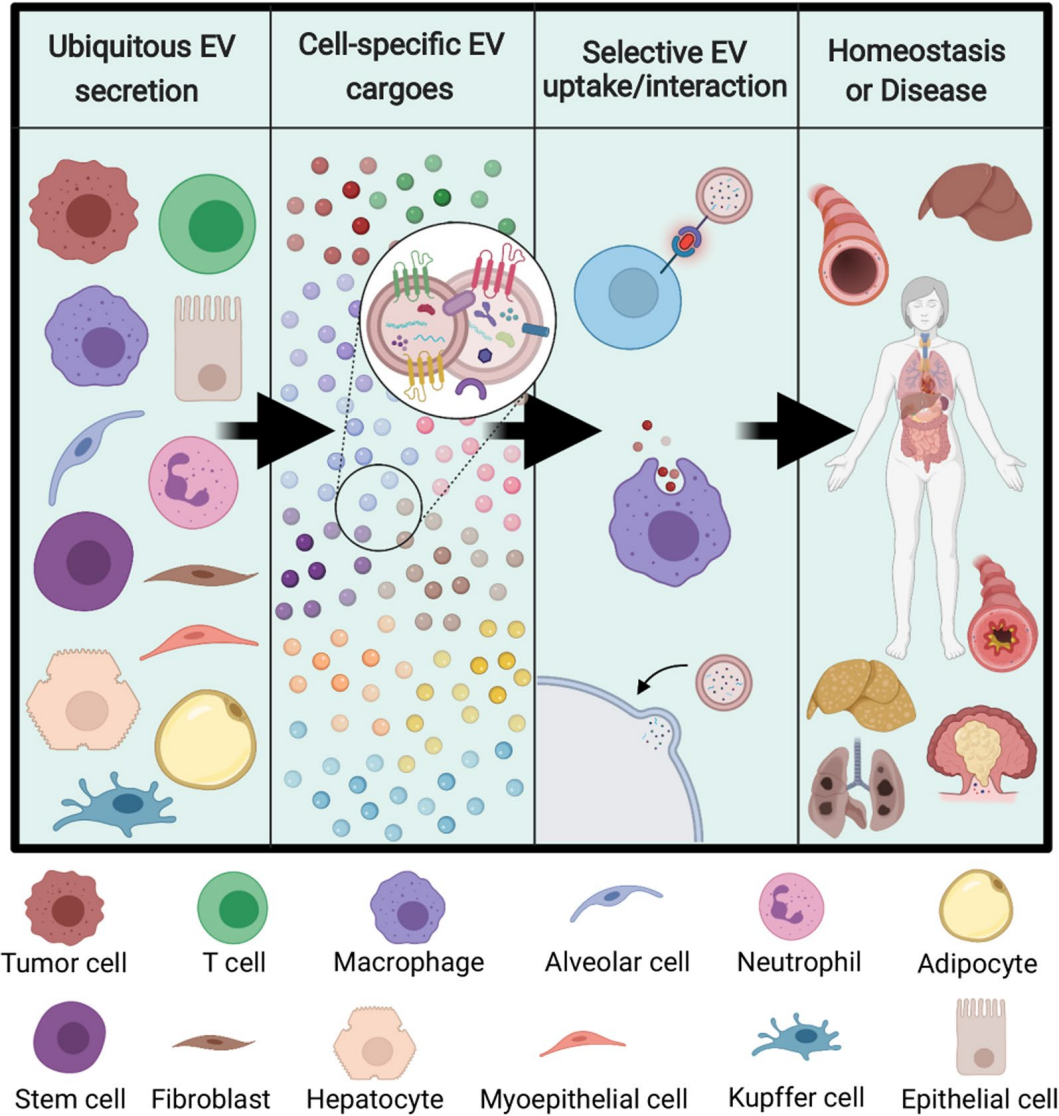
Overview of EV-mediated communication in disease progression. EV secretion is a universal process among stromal, parenchymal, immune, and cancer cells. Cargo contained in EVs will reflect the state of the parent cell, and communication via EVs will occur in a selective manner with particular recipient cells in nearby and distant tissues. Ultimately, the result of EV communication will dictate persistence of tissue homeostasis or development and progression of a disease state

Studying the biological function of extracellular vesicles (EVs) is an emerging research area, and the body of work supporting the transfer of communicating components through secreted EVs has greatly expanded. EVs represent a novel axis of intercellular communication, contributing not only to tissue homeostasis but also to the pathogenesis of immune-mediated diseases. EVs are spherical, lipid-bilayer delimited structures that are universally secreted by nearly all cell types and organisms. While EVs are subcategorized into several groups based on their biogenesis pathway, the term *extracellular vesicle* generally refers to particles in the range of 50–5000 nm, with exosomes and microvesicles (MVs; also known as microparticles) being the most commonly studied types of EVs. Exosomes are formed through the inward budding of the multivesicular body, an intracellular component of the endocytic pathway, and are typically 30–150 nm in diameter. In contrast, MVs are formed through outward budding of the plasma membrane and are generally 100–1000 nm in diameter. Exosomes and MVs overlap in size and density and share many of the same biosynthesis components, making them experimentally difficult to distinguish. Therefore, throughout this review, we will refer to exosomes and MVs as EVs. While EVs were initially believed to be cellular waste products, their status as key players in intercellular communication is now broadly recognized [1]. Cells can transfer messages to each other through EVs by sending bioactive cargo, including proteins (enzymes, surface receptors, signaling proteins), nucleic acids (mRNAs, microRNAs, DNA fragments), lipids (sterols, phospholipids, sphingolipids), and metabolites (amino acids, steroid hormones, TCA cycle intermediates) [2, 3]. Additionally, EVs have been shown to possess intrinsic, cell-specific homing capabilities [4, 5]. The striking targeting specificity of EVs is believed to be driven by distinct vesicular surface proteins [6]. These dynamic features accentuate their potential as the primary drivers of crosstalk between cells in the local microenvironment and in distant sites to contribute to tissue fates (Fig. 1).

While much evidence is accumulating regarding the importance of EVs as effective communicators, much less has been studied regarding the role of EVs in the development of pathological conditions. Recent insights gained from the experimental study of EVs derived from human subjects, tissue culture, and animal models are shaping our understanding of the cellular communication patterns that unfold during disease progression. As tissue damage develops, the information that is transmitted between stromal, immune, and dysfunctional cells dictates pathogenesis and outcome. This review serves to highlight recent work that uncovers factors and signaling pathways that are involved in EV-mediated communication within injured and diseased environments. It should be noted that EVs have been implicated in many diverse pathologies, including cardiovascular, renal, and musculoskeletal diseases [7–9]. However, in this review we highlight non-communicable disorders that involve crosstalk between immune cells and soft tissue environments. We focus on cancer, metabolic disorders, and inflammatory lung diseases given their high incidence worldwide, and the importance of the immune system in the fate of the disease [10–13]. We also provide insight into EV-mediated communication patterns in these conditions and highlight future therapeutic directions.

## EV-driven communication in the tumor microenvironment

### Immune suppression and modulation

Tumor cells interact with the immune system throughout all stages of disease development, and crosstalk between tumor cells and immune cells is critical to cancer progression [14]. Several recent studies have attributed immunomodulatory functions to cancer cell-derived EVs. Namely, the vesicular transfer of microRNAs (miRNAs)—small non-coding RNAs that are involved in the post-transcriptional inhibition and regulation of gene expression—is a key process in cancer immunomodulation. For example, colorectal cancer (CRC) cells have been shown to release EVs containing miRNA 1246, which when internalized by macrophages, caused them to undergo reprogramming and release anti-inflammatory and tumor supportive factors [15]. Macrophages play a significant role in the tumor microenvironment (TME) and can account for more than 50% of the tumor mass [16]. However, the high degree of macrophage presence within the TME is generally not associated with increased tumoricidal effects. Rather, clinical investigations reveal a correlation between high tumor-associated macrophage (TAM) density and a poor prognosis [17]. In general, macrophages are highly plastic and possess the ability for polarization or “activation” by environmental cues that results in functional phenotypic changes [18, 19]. Macrophages are typically characterized as “classically activated”, presenting an M1 pro-inflammatory phenotype, or “alternatively activated”, having an M2 phenotype involved in the resolution of inflammation and the initiation of tissue repair. Particularly, presence and infiltration of M2 macrophages has been linked to poor prognoses in several cancers [20–22]. Recently, Zhao and co-workers demonstrated that tumor EVs from CRC cells polarized macrophages into an M2 phenotype via transfer of miRNA 934 (miR-934) [23]. In vitro experiments verified that miR-934 targets and downregulates phosphatase and tensin homolog (PTEN), resulting in the activation of PI3K/AKT signaling, a key pathway that is normally antagonized by PTEN. Consequently, macrophages expressed higher amounts of the chemokine CXCL13, which can induce a positive feedback loop with tumor cells and enhance secretion of miR-934 through tumor-derived EVs. Establishment of this feedback loop through EVs allows for persistent crosstalk between macrophages and tumor cells that creates a favorable inflammatory environment for tumor progression and metastasis. Moreover, the microenvironment of most solid tumors contains hypoxic regions due to abnormal vasculature and the rapid proliferation of tumor cells [24, 25]. Tumor EVs from hypoxic conditions were shown to be enriched in chemokines and immunosuppressive factors, including colony stimulating factor 1 (CSF-1), chemokine ligand 2 (CCL2), and transforming growth factor beta (TGF-β), leading to macrophage chemotaxis and polarization into an M2-like phenotype [26]. Vesicular transfer of let-7a miRNA from hypoxic tumors to bone marrow-derived macrophages also promoted M2 polarization and enhanced oxidative phosphorylation activity through downregulation of the AKT-mTOR signaling pathway. Taken together, tumor-derived EVs play a key a role in the recruitment and maintenance of macrophages in the TME. Tumor cells use EVs to drive macrophages toward an M2 fate, which leads to an immunosuppressive environment that promotes cancer progression.

**Fig. 2:**
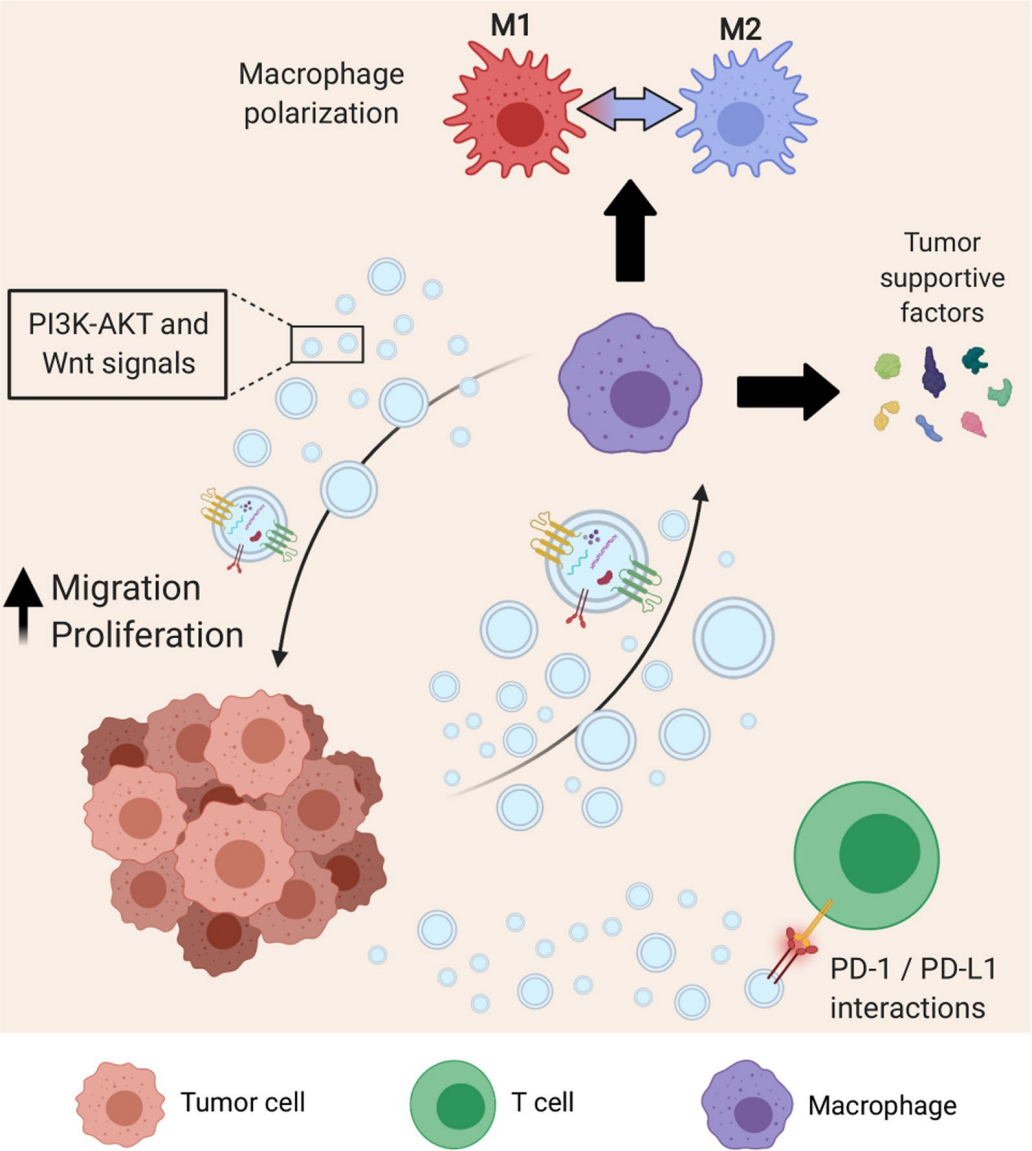
EVs in the tumor microenvironment. Tumor cells secrete EVs that target the immune system. Monocytes that uptake tumor EVs are driven toward an M2 and tumor-associated phenotype, resulting in the secretion of immunosuppressive and tumor-supportive factors. Tumor-associated macrophages release EVs that also act on tumor cells, notably through activation of P13K-AKT and Wnt signaling, causing enhanced migration and proliferation. Additionally, tumor cells evade immune surveillance by shedding EVs that contain PD-L1, which directly inhibits the T cell-mediated immune response

Within the TME, EV-mediated crosstalk is a multi-directional process. Tumor cells may reprogram and influence the behavior of immune cells, which send new information back to the tumor (Fig. 2). In the context of cancer, where a dysregulated immune response contributes to disease initiation and progression, EVs secreted from TAMs were shown to promote migration in gastric cancer cells via upregulation of PI3K-AKT signaling, resulting in cytoskeletal remodeling and enhanced migration [27]. In another study, cancer patient-derived EVs isolated from peripheral blood samples enhanced expression of vasculature endothelial growth factor A (VEGFA), Wnt5A, and interleukin 1 beta (IL-1β) in human macrophages while significantly enhancing the invasion of breast cancer cells in vitro [28]. The relevance of these factors in TME crosstalk has been explored previously, where one study showed that CRC cells can induce the secretion of IL-1β from surrounding macrophages [29]. While it was not explicitly confirmed to be encapsulated within EVs, the secreted IL-1β from these macrophages was sufficient to induce canonical Wnt signaling and promote enhanced growth in the tumor cells, despite IL-1β lacking a signal peptide that would allow it to be secreted through the canonical protein secretion pathway [30]. Thus, EV-based secretion of this cytokine, and possibly others that lack a signal peptide, such as IL-18, offers a potential mechanism of non-canonical secretion. Additionally, Wnt5a has been shown to increase the secretion of CCL2, cyclooxygenase 2 (COX-2), and prostaglandin E_2_ (PGE_2_) in macrophages, leading to the recruitment of additional macrophages and the progression of gastric cancer [31]. Due to their hydrophobicity, the extracellular diffusion of Wnt ligands is not well understood. Secretion of active Wnt ligands in EV-associated forms has been shown in vitro in drosophila and human cell cultures [32]. Ultimately, cancer cells are capable of utilizing EVs as a selective means of communication to reprogram macrophages toward a pro-tumorigenic fate, where macrophages will secrete EVs to collaborate with cancer cells for disease progression.

Additionally, tumors often adapt to exploit the immune system’s intrinsic regulatory mechanisms in order to avoid immune surveillance. One of the key immune checkpoints involves programmed cell death protein 1 (PD-1) and its ligand PD-L1 [33]. In normal biology, T cells express PD-1 and interact with PD-L1 on antigen presenting cells (APCs) to inhibit T cell activity in the late stages of an inflammatory response. It has been previously established that tumor cells may express PD-L1 intrinsically through constitutive oncogenic signaling or adaptively in response to inflammatory factors [34]. Haderk and colleagues were among the first to report PD-L1 modulation in immune cells through tumor-derived vesicles [35]. They found that EVs derived from the plasma of chronic lymphocytic leukemia patients were highly enriched in the noncoding RNA hY4 when compared to that in healthy individuals. Transfer of vesicular hY4 to monocytes was shown to involve Toll-like receptor 7 (TLR7) signaling, which induced the expression of PD-L1. Mechanistically, the same effect can be achieved through the release of PD-L1 expressing EVs from tumor cells themselves. Indeed, Chen et al. reported vesicular PD-L1 mediated immunosuppression from melanoma cells in both cell culture- and patient-derived EV samples [36]. PD-L1 + EVs were shown to be taken up by CD8 + T cells, which suppressed their proliferation, cytokine secretion, and cytotoxic capacity. Notably, similar results were observed regarding tumor-derived EVs in breast and prostate cancers [37, 38].

EVs clearly play a central role in intercellular communication between tumor and immune cells. Cancer cell-derived EVs participate in driving immune cells to adopt pro-tumor phenotypes, allowing the tumor to systemically evade immune surveillance and establish favorable microenvironments. Additionally, EVs secreted by dysregulated immune cells in the tumor microenvironment can induce changes in cancer cells, such as increased migration and proliferation, that contribute to the progression of cancer. These studies highlight the importance of EVs in mediating tumor–immune cell crosstalk in the TME.

### Effects of post-therapy tissue damage on tumor-stromal interactions

#### Chemotherapy

Chemotherapy is a widely implemented systemic therapy to treat cancer patients. Chemotherapeutic drugs generally affect rapidly proliferating cells in many parts of the body in addition to the tumor [39]. Most chemotherapeutic drugs used to treat cancer induce oxidative stress through the elevation of intercellular reactive oxygen species (ROS) levels [40, 41]. Excessive ROS levels and the accumulation of oxidized biomolecules may overwhelm a cell’s antioxidant capabilities and induce cytotoxicity [42]. Several studies have demonstrated that exogenous ROS induce the secretion of EVs in human alveolar and epithelial cells [43, 44]. In neutrophils and monocytes, treatment with CO_2_ to induce intrinsic ROS generation was also shown to result in enhanced EV secretion [45].

Recently, Shen et al. revealed an EV-mediated mechanism of chemotherapy resistance in breast cancer [46]. Cancer cells were treated with docetaxel or doxorubicin, and EVs derived from treated cells were investigated. Expression levels of several vesicular miRNAs were significantly elevated in EVs derived from cells that were treated with the chemotherapeutics, and those EVs induced cancer stem cell characteristics in naïve breast cancer cells, including enhanced sphere forming efficiency, expression of ATP-binding cassette (ABC) transporters, and a stemness-associated gene profile. Breast cancer stem cells are critically involved in tumor recurrence and metastasis and have robust therapy resistance properties [47]. EV-mediated communication of chemotherapy-injured tumor cells with the surviving tumor fraction may therefore promote broader chemoresistance in patients.

**Fig. 3:**
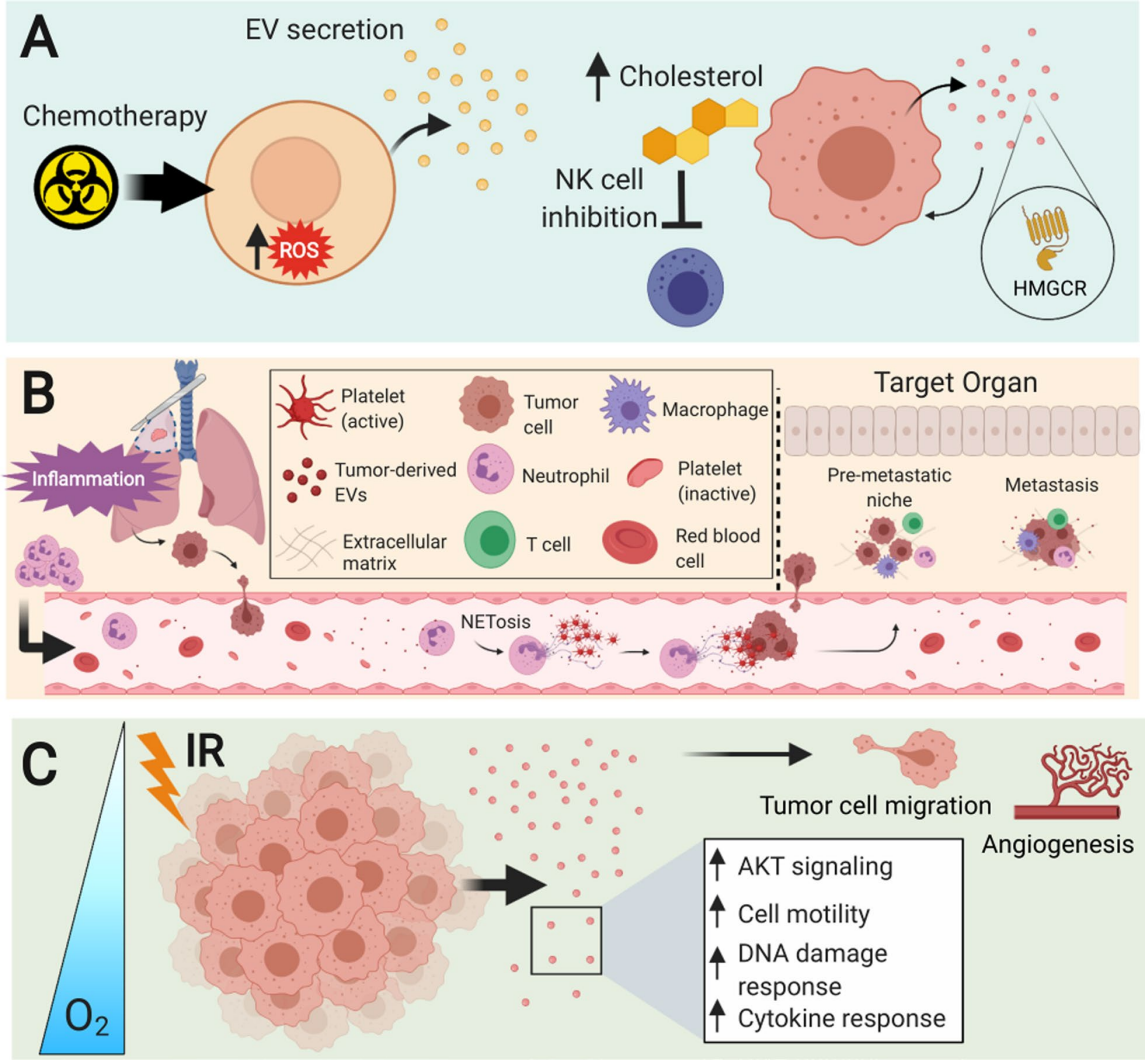
The role of EVs post-cancer therapy. **A** Chemotherapy increases intracellular levels of reactive oxygen species (ROS), which leads to higher EV secretion. EVs derived from tumors that have been exposed to chemotherapeutic drugs have been shown to contain HMG-CoA reductase (HMGCR), which acts in an autocrine/paracrine manner, resulting in a vicious cycle of cholesterol production in cancer cells. Enhanced cholesterol levels can suppress natural immunity through inhibition of NK cells. **B** Surgical manipulation of tumors may induce circulating tumor cell (CTC) generation while causing an inflammatory state characterized by increased neutrophils in circulation. Tumor-derived EVs in circulation interact with neutrophils to induce NETosis, which allows CTCs to reach target organs. Tumor cells and tumor-derived EVs, along with immune cells, establish a pre-metastatic niche at this site. **C** After tumor cells have been exposed to ionizing radiation (IR), EV secretion is enhanced with cargo contents involved in AKT signaling, cell motility, DNA damage, and cytokine responses. Under hypoxic conditions, EV secretion is further increased, and these EVs promote migration in tumor cells and angiogenesis

Chemotherapy-induced alterations in the relationship between EVs and cholesterol transfer was recently studied in acute myeloid leukemia (AML) [48]. AML cells were shown to increase cholesterol levels and secrete a significantly greater number of EVs containing HMG-CoA reductase (HMGCR) after treatment with cytarabine. Furthermore, AML EVs were involved in autocrine signaling, which resulted in increased cholesterol production and cellular proliferation in recipient AML cells. This elucidates a vicious cycle of cholesterol production that is dependent upon EV-mediated autocrine signaling (Fig. 3A). High levels of cholesterol could negatively regulate the immune system, for example thorough inhibition of sterol regulatory element-binding proteins in NK cells, leading to dysfunction in anti-tumor immunity and development of chemoresistance [48, 49].

#### Surgery

The majority of cancer patients will undergo surgery as part of their therapy. Patients with locally controlled solid tumors are more likely to receive surgery as their sole or primary form of treatment [39]. Evidence from clinical studies has shown that surgical manipulation of various tumors induce dissemination of circulating tumor cells (CTCs) [50]. Moreover, Fu et al. demonstrated that EVs containing SMAD3 protein and mRNA derived from hepatocellular carcinoma cells enhanced the adhesion capabilities of tumor cells in vitro while also showing the uptake of tumor EVs by CTCs and increased metastatic burden in vivo [51]. These findings highlight the EV-mediated communication between primary tumors and CTCs, establishing a mechanism through which primary tumor cells aid in the survival of CTCs and in their ability to adhere to distant metastatic sites. Additionally, the ability of tumor-derived vesicles to form pre-metastatic niches in distant tissues has been described. For instance, macrophage migration inhibitory factor (MIF) containing EVs from pancreatic cancer cells were shown to selectively target and activate Kupfer cells (KCs) in the liver, resulting in increased secretion of TGF-β [52]. This led to enhanced fibronectin production by hepatic stellate cells and subsequent macrophage infiltration. Macrophage infiltration has been shown to be positively correlated with metastasis and pre-metastatic niche formation in cancer patients and mouse models [53, 54]. Furthermore, Zeng et al. showed that vesicular miR-25-3p transferred from CRC cells to endothelial cells increased vascular permeability by targeting Krüppel-like Factor 2 and 4 (KLF2/4) [55]. With enhanced permeability, CTCs can cross the endothelial barrier and extravasate into surrounding tissues, which may lead to metastatic disease.

Complications arising from CTC dissemination are further compounded when considering the role of surgery-induced immune activation. Surgical injury results in the activation of immune cells, such as monocytes, lymphocytes, and granulocytes, and enhances the postoperative inflammatory environment [56]. While the immune response after surgery is imperative in the wound healing process, many patients undergoing major oncological surgeries can develop systemic inflammatory response syndrome (SIRS) that may last up to one week or more [57, 58]. Additionally, acute inflammatory conditions such as SIRS are typically associated with the hyperactivation of neutrophils, which are the most abundant type of circulating white blood cell and have long been implicated in the immune response to surgery [59–61]. Brinkmann et al. first demonstrated that neutrophils could release granule proteins and nuclear chromatin to generate neutrophil extracellular traps (NETs), which are structures that can entangle and eliminate pathogens extracellularly [62]. Park and colleagues established a connection between cancer cells, NETs, and metastasis, where they showed that breast cancer cells induced metastasis-supporting NET formation while treatment with DNase I-coated nanoparticles attenuated lung metastases [63]. Cooperation between tumor-derived EVs and neutrophils in tumor progression was recently proposed by Leal et al*.,* whose work has suggested that granulocyte colony-stimulating factor (G-CSF) from tumor cells induces activation and release of neutrophils into the circulation, where interaction with tumor-derived EVs promotes the release of NETs [64]. To our knowledge, this has been the only study to examine the interactions between tumor-derived EVs and neutrophils with respect to NETosis—the release and development of NETs. However, several factors that are involved in NET formation have been shown to be involved in EV-mediated communication. Tissue factor (TF) is critical to the coagulation cascade, and TF-decorated NETs have been shown to be released by neutrophils under inflammatory conditions, resulting in platelet activation that further promotes NETosis [65]. In a mouse model, pancreatic cancer cell-derived EVs were shown to express TF and bind avidly to NETs, contributing to thrombosis [66]. While cell interactions were not explicitly investigated, it is possible that TF-containing EVs released from a primary tumor may interact with circulating neutrophils and platelets and contribute to NETosis. An overview of the cascade connecting surgery-induced inflammation and cancer metastasis is shown in Fig. 3B.

#### Radiotherapy

Radiation therapy is an integral component of palliative and curative care for cancer patients, with estimates that over 50% of patients will receive radiation as part of their treatment [67]. Ionizing radiation (IR) has been established as a factor that significantly impacts intercellular communication. Nagasawa and Little were among the first to propose that genetic changes in cells that are not directly irradiated result from altered intercellular signaling [68]. Termed radiation-induced bystander effects (RIBE), these biological changes in cells outside of the direct field of radiation may be capable of causing systemic effects [69]. Recently, there has been a desire to determine a connection between EV-mediated communication and RIBE. Radiotherapy exposes both healthy tissue and tumor cells to IR, causing DNA damage in both [67]. Yu and colleagues first reported enhanced EV production in cells undergoing a DNA damage-induced stress response [70]. From their work analyzing the treatment of lung cancer cell lines with 5 Gy of γ irradiation, they postulated that IR-induced DNA damage leads to activation of the transcription factor p53, allowing for the upregulation of tumor suppressor-activated pathway 6 (TSAP6). TSAP6 has been identified as a key player in EV secretion as radiation-induced secretions of EVs were abrogated in TSAP6-null mice [71]. Additionally, more recent studies have reproduced the observation of radiation-induced enhanced EV secretion in head and neck and breast cancer models [72, 73].

Furthermore, the factors transferred by EVs in this context have recently been highlighted as multiple studies have reported changes in the composition of EVs following IR. Several groups have analyzed changes in vesicles secreted from head and neck cancer cells following moderate doses of in vitro radiation. Mutschelknaus et al. found nearly 80 differentially regulated proteins in EVs isolated from cells treated with 6 Gy of radiation [74]. Protein function analysis revealed that the deregulated proteins were primarily involved in cell motility and AKT signaling, and treatment of cancer cells with the isolated EVs ultimately resulted in increased matrix metalloproteinase (MMP)-2 and MMP-9 expression. Similarly, Abramowicz and colleagues analyzed the miRNA profile of EVs derived from irradiated head and neck cancer cells treated to doses of up to 8 Gy and found that the differentially expressed miRNA species after radiation were linked to genes involved in cytokine-mediated and DNA damage responses [75].

Radiation-induced alterations in EV signaling are believed to be influenced by oxygen levels in the environment, but this relationship is poorly understood. As portions of the TME are often hypoxic, it is important to understand how IR further compounds changes in cellular crosstalk. Jung and colleagues investigated EV dynamics under normoxic and hypoxic conditions and with or without IR treatment in a breast cancer model [76]. They reported higher levels of EVs derived from cells under hypoxic conditions, with the largest amount of vesicles secreted by irradiated hypoxic cells. Additionally, EV uptake by hypoxic breast cancer cells was found to be higher when the EVs were secreted from hypoxic or irradiated cells, and the highest uptake was achieved for hypoxic non-irradiated EVs. Similarly, Mo et al. recently published their findings regarding the analysis of lung cancer-derived EVs [77]. EVs from hypoxic irradiated cells most prominently enhanced cell migration and invasion in cancer cells while leading to increased proliferation and vascularization in endothelial cells. Furthermore, proteomic analysis showed that vesicular angiopoietin-like 4 (ANGPTL4) was a key factor. The interplay between IR-induced damage and the oxygen levels within the TME appear to impact EV-mediated communication (Fig. 3C).

While studies regarding radiation-induced modifications of EV crosstalk have focused primarily on the response of cancer cells, the EV-mediated radiation response in normal tissue cells has yet to be significantly explored. As normal tissue irradiation has been implicated in cancer recurrence under immunocompromised conditions, EV-mediated tumor–stromal interactions following IR will be imperative to study to more comprehensively understand the effects of IR-associated changes in EV composition on intercellular communication and disease progression [54].

## The role of EVs in the progression of metabolic disorders

### Cell–cell communication in obesity and diabetes

Metabolic disorders, generally characterized by hyperglycemia, hyperlipidemia, hypertension, and high body-fat, are estimated to affect about one-fourth of world’s population [78]. Moreover, the World Health Organization has described obesity as a “global epidemic”, with the rate of obesity in adults in the United States reaching levels over 40% [79, 80]. Circulating levels of EVs are enhanced in patients with disorders such type 2 diabetes, dyslipidemia, and obesity [81]. A recent in vivo study revealed that mice with adipose-specific knockdown of Sirt1 experienced excessive fat accumulation, disordered glucose metabolism, insulin resistance, and increased EV secretion [82]. However, treatment with the EV inhibitor GW4869 reversed the impaired metabolic profile in the mice. These results highlight a relationship between Sirt1 and EV-mediated communication in adipose tissue. While the underlying mechanism was not explored in depth, the authors found that insulin sensitivity was modulated by EVs at least partially via the TLR4/NF-κB signaling pathway. Additionally, adipose tissue has been shown to be a major source of miRNA-carrying EVs [83]. In another study, adipocytes in obese adipose tissue were found to release EVs carrying miR-34a, which was transferred to resident macrophages and caused a shift from an M2 to M1 phenotype [84]. This came as a result of the downregulation of the transcription factor KLF4 and led to a highly inflammatory and fibrotic environment, thereby leading to systemic glucose intolerance and insulin resistance. Another study found that EVs from adipose tissue macrophages (ATMs) were key players in mediating insulin resistance. Ying et al. found that miR-155 was enriched in obese ATM EVs, and that systemic treatment of lean mice with obese ATM EVs resulted in glucose intolerance and insulin resistance [85]. While this study analyzed communication through adoptive transfer of EVs, it provides evidence for the cross talk between ATMs and cells in other tissues such as the liver, visceral adipose tissue, and muscle.

**Fig. 4:**
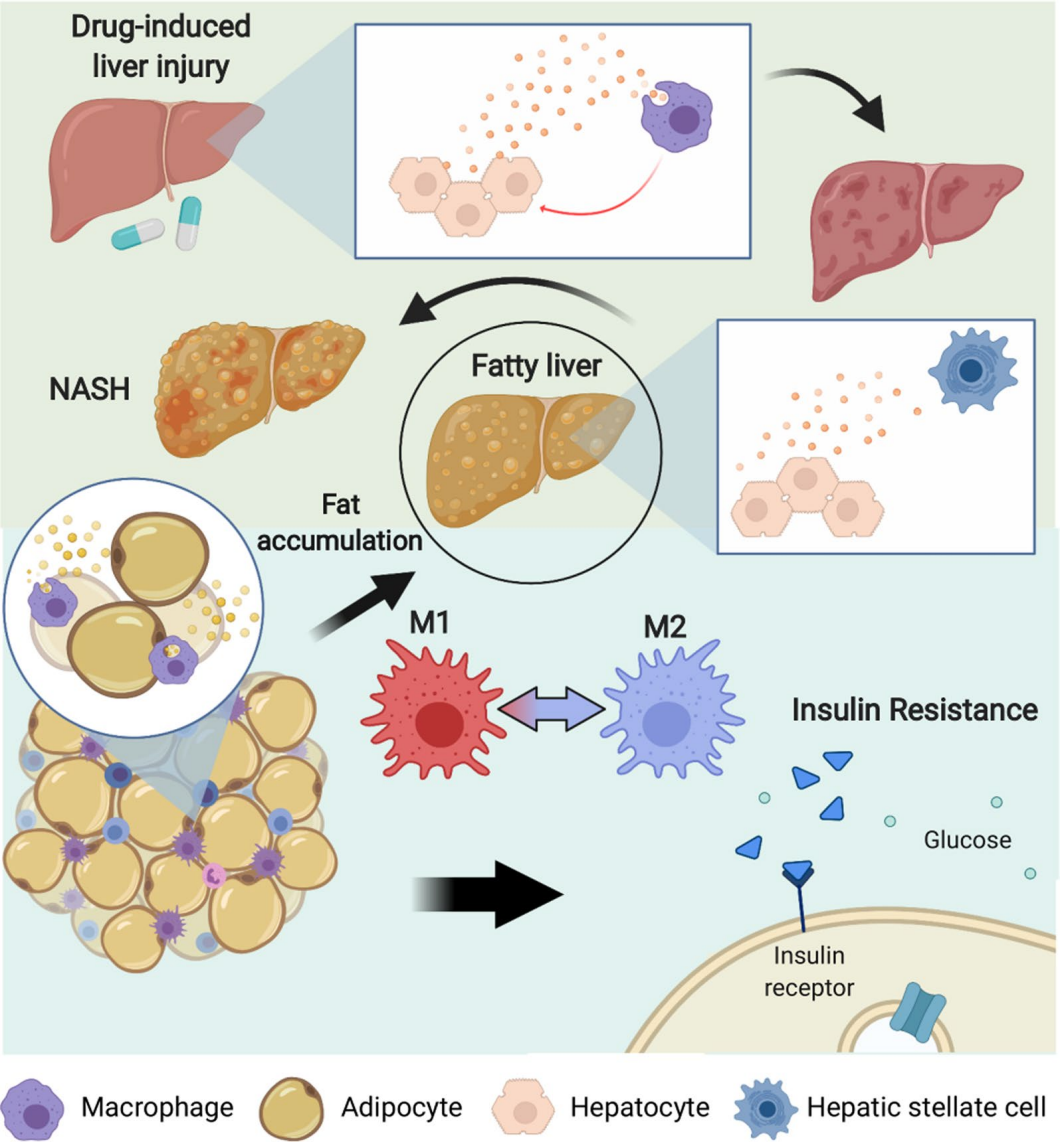
Overview of EV-mediated communication in liver diseases. In drug-induced liver damage, EV communication between hepatocytes and macrophages induces an inflammatory and fibrotic state. Furthermore, accumulation of fat in the liver, which is often associated with obesity, leads to communication between hepatocytes and hepatic stellate cells that promotes progression of fatty liver to non-alcoholic steatohepatitis (NASH). Within adipose tissue of obese individuals, adipocytes communicate with macrophages to polarize them into an M2 phenotype and create an inflammatory environment that leads to insulin resistance

Freeman and colleagues recently revealed that patients with diabetes had higher levels of circulating EVs compared to euglycemic individuals [86]. Furthermore, the level of insulin resistance in patients was positively correlated with plasma EV concentrations while prolonged insulin treatment in vitro similarly resulted in elevated EV secretion in primary neuronal cells. EVs from diabetic patients were preferentially taken up by circulating leukocytes, namely monocytes and B cells, when compared to EVs from euglycemic controls, which altered the gene expression of the recipient cells. These results suggest a connection between insulin resistance and EV secretion in human diabetic patients while also highlighting the enhanced communicative ability with immune cells. While the precise ligands that contributed to enhanced uptake of diabetic EVs were not explored, a controlled shift in EV-mediated communication patterns is evident. Therefore, the interactions between the immune system and several tissues and organs in the body, including muscle and adipose tissues, the pancreas, and the liver, are imperative to the onset and progression of diabetes. An overview of the relation between fat accumulation, EV communication, and disease progression is shown in Fig. 4.

### EV regulation of liver diseases

The liver plays an essential role in metabolism and the immune system. Within the liver, hepatocytes can send metabolically active enzymes via EVs. Angulo et al. recently reviewed the current status of active enzyme-carrying EVs in liver conditions [87]. Notably, active arginase 1 has been detected in EVs derived from rat hepatocytes as evidenced by a substantial change in L-arginine levels after adding EVs isolated from acetaminophen-treated cultures to serum [88]. Dysregulated arginine breakdown has serious implications in processes like vascularization, which is regulated by the metabolism of arginine. Drug-induced damage to liver cells may in turn propagate abnormal metabolic processes in distant tissues through EV-mediated communication. Previous work shows that early drug-induced liver injury events are initiated by hepatocyte-derived EVs. Using an in vivo rat model, Holman et al. found that plasma levels of vesicular miR-122, which is a highly enriched miRNA in the liver, decreased over time while albumin mRNA increased when rats were treated with subtoxic acetaminophen doses [89, 90]. Since there were no significant changes in the number of EVs, the packaging of these RNA species appears to be a selective means of communication, even in the absence of overt liver injury. Moreover, hepatocyte transfer of miR-122 via EVs to human monocytes has been shown to sensitize monocytes to lipopolysaccharide (LPS) treatment and induce an enhanced inflammatory response that is characterized by increased tumor necrosis factor alpha (TNF-α) and IL-1β production [91]. An augmented inflammatory state such as this is pivotal in the development of alcohol and drug-induced steatohepatitis [92]. Ibrahim and colleagues revealed a similar interaction between liver EVs and the immune system. They found that lipotoxic treatment of hepatocytes resulted in the enrichment of CXCL10 in EVs, an effect that was attenuated when mixed lineage kinase 3 (MLK3) was knocked out or pharmacologically inhibited [93]. Later work by Ibrahim and colleagues showed that the enhanced CXCL10 in EVs was specifically associated with pro-inflammatory macrophage infiltration in this context [94]. These results suggest that liver cell lipotoxicity, which occurs with high accumulation of fat in the liver, induces EV-mediated recruitment of non-resident macrophages that sets the stage for disease progression in the liver.

The worldwide prevalence of nonalcoholic fatty liver disease (NAFLD) is increasing, with recent estimates claiming roughly 1 in 4 individuals worldwide are afflicted with NAFLD [95]. These rates will likely continue to rise as the global obesity epidemic continues to develop. Dysregulated lipid metabolism leads to the accumulation of excess fat in the liver, causing hepatic steatosis. Lee et al. demonstrated that palmitic acid (PA)-treated hepatocytes significantly increased the production of EVs. These EVs contained a distinct miRNA profile compared to untreated cell-derived vesicles, including substantially higher levels of miR-122 and miR-192 [96]. Additionally, hepatic stellate cells (HSCs) incubated with EVs from PA-treated hepatocytes had increased expression of various fibrotic markers, including alpha smooth muscle actin (α-SMA), TGF-β, and alpha-1 type I collagen (Col1a1). Notably, a fibrotic environment and the accumulation of macrophages are characteristics of nonalcoholic steatohepatitis (NASH), a more severe form of liver disease [97]. The progression from steatosis to NASH may depend on EV-mediated communication between hepatocytes and HSCs. There are no approved therapies for NASH, and thus understanding the processes involved in its progression will be imperative for the development of novel treatments.

## EVs in inflammatory lung diseases

**Fig. 5:**
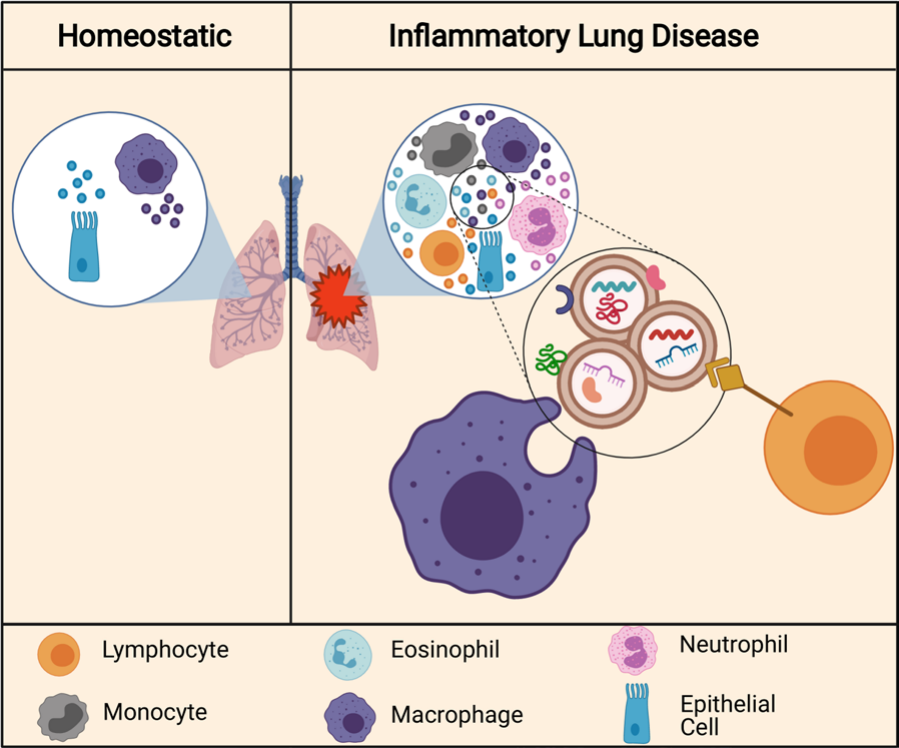
EVs in inflammatory lung disease. In homeostasis, most EVs in the airway are secreted by epithelial cells lining the airways and alveolar macrophages. In inflammatory lung diseases, immune cells are recruited to the airways and contribute to EV secretion. Secreted EVs carry protein, lipid, and miRNA cargoes and can be taken up by other immune cells and epithelial cells in the local environment. These EVs and cargoes can induce pro-inflammatory or anti-inflammatory effects in target cells, depending on the cargo type and context. These EVs can also induce tissue damage and fibrosis

Emerging evidence has shown that immune cell-derived EVs play a critical role in the pathology of inflammatory lung diseases, including asthma, chronic obstructive pulmonary disease (COPD), and acute lung injury/acute respiratory distress syndrome (ALI/ARDS). Airway lining fluid, which is sampled by bronchoalveolar lavage, is highly populated by EVs [98]. EVs found in this fluid are derived primarily from mucosal epithelial cells lining the airways and are likely an integral component of the local airway tissue [99]. Not only are EVs present in the airways during homeostasis, they are increased in the airway lining fluid of mice with lung inflammation caused by both asthma and infection [99, 100]. Enhanced EV levels have also been found in the airways of human asthmatic patients [101]. In addition to an increase in number, the composition of EVs differs between healthy and inflamed airways. The frequency of major histocompatibility complex class II and CD54 carrying EVs is higher in the bronchoalveolar lavage fluid (BALF) of asthmatic individuals compared to that in healthy individuals [101]. EVs isolated from the BALF of individuals exposed to secondhand smoke were also shown to have lower abundance of the lipid ceramide than those isolated from individuals unexposed to secondhand smoke [101]. Additionally, immune cell-derived EVs are increased in the airways of mice with induced allergic airway inflammation compared to control conditions [99]. The fact that EVs are increased and have different compositions in inflammatory lung diseases indicates that they may be involved in pathogenesis. Figure 5 shows an overview of the immune cells that are recruited to the airways in inflammatory lung disease.

### Pro-inflammatory processes

In addition to being increased in biofluids in response to inflammatory stimuli, EVs carry critical communicating cargoes that implicate them in immune-mediated pathology. In fact, EVs in the lung have been demonstrated to carry cytokines that play critical roles in the communication between immune cells. For example, in mouse models of infection-exacerbated asthma and COPD, EVs secreted into the airway carry the pro-inflammatory cytokines IL-18 and IL-1β, resulting in airway lining fluid and lung tissue neutrophilia [100]. In mouse models of ALI, macrophages and neutrophils were found to secrete EVs carrying the pro-inflammatory cytokine TNF-α following induction of lung injury by airway LPS instillation [102, 103]. Moreover, EVs derived from alveolar macrophages primed in vitro with an ALI-inducing stimulus were shown to induce ALI hallmarks, including airway neutrophilia, when instilled into the airways of naïve mice [102]. These studies suggest that immune-derived EVs contribute to the pathology of inflammatory lung diseases through their cytokine cargoes. This is consistent with in vitro work demonstrating that immune cells secrete cytokine-laden EVs in isolation [104–106].

Apart from cytokines, reports also show that immune cell-derived EVs carry active enzyme cargoes. Macrophages, dendritic cells, and neutrophils secrete EVs carrying leukotriene biosynthesis enzymes while EVs secreted by activated T cells have been shown to carry the TCR-associated kinase Lck [107–110]. This supports the idea that EVs play integral functional roles in the environment of the airway tissue, perhaps by targeting functional enzymes to locations where substrates are present or by protecting enzymes from degradation and inactivation. In the context of COPD, EVs secreted by activated neutrophils carry, protect, and target the enzyme neutrophil elastase, which degrades extracellular matrix (ECM) components and contributes to COPD pathogenesis [111]. Exposure to tobacco smoke also induces macrophages to secrete EVs containing proteolytically active MMP-14 in mice [112]. In addition, EVs derived from the airway lining fluid of asthmatic patients have been shown to increase leukotriene biosynthesis in recipient cells [109]. Under ALI/ARDS conditions, lung epithelial cells communicate with immune cells by releasing EVs containing enzymes, including prolyl endopeptidase involved in the generation of the collagen-derived neutrophil chemoattractant Pro-Gly-Pro and caspase-3, which activates alveolar macrophages [113, 114]. These studies suggest that immune cell-derived EVs enhance and extend immune cell functions by delivering active enzyme cargoes.

Immune cell-derived EVs in the lung have also been shown to carry nucleic acids, where the miRNA content of EVs has been the subject of extensive study. Extracellular miRNAs derived from immune cells are increased in the airway lining fluid of mice with induced allergic airway inflammation, and these miRNAs are likely packaged into EVs as they are protected from degradation by RNases [99]. This indicates that immune cells secrete miRNA-laden EVs into the airways in response to inflammatory stimuli and that packaged miRNAs could serve to transmit inflammatory signals. In support of this concept, EVs secreted by epithelial cells in models of ALI contain miRNA species capable of inducing macrophage activation and recruitment into the lungs [115, 116]. Additionally, in a mouse model of ARDS, miR-466 family miRNAs were released into the airways in EVs where the RNA cargoes activated the NLRP3 inflammasome, a protein complex required for the release of pro-inflammatory cytokines such as IL-1β in macrophages [117]. Immune cell-derived EVs carry a variety of bioactive cargoes capable of contributing to the pathogenesis of inflammatory lung diseases by facilitating intercellular communication. EVs may serve to transport and target immunomodulatory molecules like cytokines, active enzymes, and microRNAs that alter the behavior of target cells and tissues, ultimately contributing to the pathology of inflammatory lung diseases.

Owing to the difficulty of identifying the active EV components that exert a biological function on target cells, many studies have identified possible roles for EVs in inflammatory lung diseases without identifying a molecular mechanism. For example, Cañas and colleagues found that eosinophils isolated from the peripheral blood of asthmatic patients, but not healthy controls, secrete EVs that induce apoptosis in epithelial cells and inhibit wound healing in vitro [118]. This suggests that EVs derived from eosinophils may be involved in the airway remodeling characteristic of asthma. Additionally, it has been found that EVs isolated from the BALF of mice with ARDS/ALI contribute to macrophage recruitment to the airways and to the regulation of cytokine production and TLR expression in alveolar macrophages [119]. These EVs were derived primarily from airway epithelial cells when the inducing stimulus was sterile and from alveolar macrophages when the stimulus was infectious [119]. This indicates that cells in the airway produce EVs in response to specific inflammatory signals and that these EVs contribute to ARDS/ALI inflammation. Additional studies are required to uncover the factors and molecular mechanisms that govern these effects.

### Anti-inflammatory processes

In addition to their pro-inflammatory effects, immune cell-derived EVs can also play anti-inflammatory and regulatory roles in pathologic lung inflammation. Alveolar macrophages secrete EVs containing suppressor of cytokine signaling 1 (SOCS1) and SOCS3, which are proteins that inhibit inflammatory cytokines involved in the JAK/STAT3 pathway [120]. These suppressive EVs are taken up by epithelial cells and act to inhibit the activation of the JAK/STAT3 pathway. When alveolar macrophages are treated with cytokines produced in allergic inflammation, they no longer release suppressive EVs [121]. This evidence indicates a mechanism by which inflammation negatively regulates a critical axis of communication mediated by EVs that maintains homeostasis in the airways. EVs isolated from the lungs of mice have also been shown to contain the anti-inflammatory cytokines TGFβ-1 and IL-10. These EVs likely originate from dendritic cells that can communicate with T cells to inhibit their proliferation in response to a specific antigen [122]. There is also evidence that immune cell-derived EVs are involved in tissue remodeling and wound repair after inflammation has resolved, which can be pathological in the development of fibrosis in the lungs. M2 macrophages secrete EVs containing miR-328, which regulates the expression of FAM13A in fibroblasts to promote pulmonary fibrosis [123]. Likewise, it has been found that airway epithelial cells may transfer inositol polyphosphate 4-phosphatase to fibroblasts, increasing proliferation and contributing to lung fibrosis following airway inflammation [45]. Thus, immune cell-derived EVs are also implicated in anti-inflammatory intercellular communication in the lung, which may become pathological when EVs are involved in dysregulated wound responses.

## Conclusions and future perspectives

EVs serve as vital mediators of intercellular communication between immune, parenchymal, and stromal cells in tissues. As such, it is intriguing to consider how the information provided by EVs may be harnessed to diagnose and treat a wide range of diseases. Many studies have investigated the use of specific cargo components in EVs derived from biological fluids to use as biomarkers for disease detection. For example, EVs with particular lipid and miRNA cargoes, such as ceramides and miRNAs with target transcripts involved in cytokine and MAPK signaling, are altered in airway lining fluid collected from asthmatics compared to healthy patients [101, 124, 125]. Many of the studies reviewed here provide additional support for using EV components as biomarkers (Tables 1 and 2).

**Table 1:** Cell types and biomolecules involved in EV-mediated disease progression

Disease	EV-secreting cell type	EV cargo changes	Effect on progression	References
CRC	Tumor cells	↑ miR-1246	Causes macrophage shift to anti-inflammatory and M2 phenotype	[8, 11]
		↑ miR-934		
Melanoma	Tumor cells	↑ CSF-1	Macrophage chemotaxis and polarization to M2-like phenotype	[26]
		↑ CCL-2		
		↑ TGF-β		
CLL	Cancer cells	↑ hY4	Induces expression of PD-L1 in monocytes	[35]
Melanoma	Tumor cells	PD-L1 expression	Suppression of proliferation, cytokine secretion, and cytotoxic abilities in CD8 + T cells	[36]
NAFLD	Hepatocytes	↑ miR-122	Sensitizes macrophages to lipids, inducing enhanced inflammatory response	[73, 75, 80]
		↑ miR-192		
Obesity	Adipocytes	↑ miR-34a	Uptake by macrophages, leading to shift to M2 phenotype; glucose intolerance and insulin resistance	[82]
NASH	Hepatocytes	↑ CXCL10	Recruitment of macrophages to the liver	[80, 81]
COPD	Neutrophils	↑ Neutrophil elastase	Degradation of ECM	[111]
ALI/ARDS	Epithelial cells	↑Prolyl endopeptidase	Neutrophil chemoattraction and macrophage activation	[113, 114]

**Table 2:** Cell types and biomolecules involved in EV-mediated progression of tissue injury

Injury-causing agent	EV-secreting cell type	EV cargo changes	Effect on injury response	References
Docetaxel, doxorubicin	Breast cancer cells	↑ miR-203a-3p	Induction of cancer stem cell characteristics	[46]
		↑ miR-9-5p		
		↑ miR-195-5p		
Cytosine arabinoside	AML cells	↑ HMGCR	Increased cholesterol production and proliferation in AML cells	[48]
IR (6 Gy)	Head and neck cancer cells	↑ FGFR1	Enhanced migration and invasion in cancer cells; enhanced MMP-2 and MMP-9 expression	[74]
		↑ HSP90		
		↓ KRT80		
		↓ LAMP1		
IR (3 Gy) + hypoxia	Lung cancer cells	↑ ANGPTL4	Promotes migration and invasion in cancer cells; increased vascularization in endothelial cells	[77]
Tobacco smoke	Macrophages	↑ MMP-14	Degradation of lung connective tissue in emphysema	[112]

### Metabolic diseases

Beyond use as biomarkers, knowledge of EV-mediated communication may be directly applied to the treatment of tissue injuries and immune-mediated diseases. Targeting the interactions between parenchymal cells and immune cells may result in favorable outcomes that attenuate disease progression. In liver diseases, EV-mediated transfer of miR-122 has been implicated in the development of an inflammatory state that is critical to steatohepatitis development [89, 91, 96]. Thus, vesicular miR-122 derived from hepatocytes is an attractive therapeutic target. However, systemic effects regarding this level of disruption remain to be fully understood. In vivo work by Ibrahim et al. demonstrated that MLK3 deficiency diminished liver injury and inflammation [93], indicating that targeting MLK3 pharmacologically in hepatocytes also has substantial potential. Additionally, anti-CXCL10 monoclonal antibody (mAb) therapy has been used in clinical trials for other conditions and could be implemented in NASH [126, 127]. Using mAb-based therapies for blockade of EV-specific components has yet to be explored and poses significant technical challenges. Furthermore, Adipocytes also communicate with macrophages to induce M2-polarization in obese conditions [84]. Site-specific blocking of EV-communication within adipose tissue through targeted use of GW4869 could have immense benefits towards limiting the development of insulin resistance. Additionally, adoptive transfer of EVs from lean mice has been shown to result in near normalization of glucose tolerance and improvements to systemic insulin sensitivity [85]. Thus, further investigations into the use of EVs as an adoptive transfer therapy could provide beneficial outcomes.

### Cancer and cancer therapies

Processes involving polarization of naïve or pro-inflammatory M1 macrophages to an anti-inflammatory M2 phenotype are appealing targets for novel immunotherapies. Tumor cells in the TME rely heavily on polarization of macrophages to create a pro-tumor and immunosuppressive environment [23, 26]. Furthermore, a phase I clinical trial is investigating the use of an antisense oligodeoxynucleotide against insulin-like growth factor type I for treatment of malignant gliomas. The antisense molecule, released from a small implanted diffusion chamber that induces apoptotic cell death in surrounding tumor cells, is hypothesized to work together with tumor-associated antigen carrying EVs released from apoptotic cells to stimulate an anti-tumor immune response in the TME [128].

Regarding cancer therapies, several strategies may be explored to target EV-mediated communication in an effort to maximize the efficacy of traditional therapies and eliminate therapy-induced recurrence. Improvements to oncological surgeries through endoscopic techniques may allow fewer CTCs to enter the circulation, which may reduce the likelihood of interaction with NETs and primary tumor-derived EVs in circulation and limit the formation of metastatic niches. Another key focus should be restricting interactions with primary tumor-derived EVs and neutrophils. Targeting EV–neutrophil interactions is a challenge, but blocking TF-positive EVs through mAb-based therapies is one such option. Much work remains to be done to elucidate the connection between radiotherapy-injured tissues and EV-mediated communication that results in cancer recurrence. Radiotherapy could be improved by enhancing normal tissue radioprotection such that EV release and dysregulated communication do not result from IR-induced DNA damage. Protecting normal tissue cells from ROS damage induced by chemotherapy may also abrogate abnormal EV-mediated communication.

### Inflammatory lung diseases

In inflammatory lung diseases, EVs derived from immune cells may be manipulated to serve as couriers of molecules that have therapeutic effects when delivered to their target cells. This has been explored through the development of EVs decorated with chimeric antigen receptors (CARsomes). CARsomes derived from dendritic cells were shown to induce apoptosis in antigen-specific T helper 2 (T_h_2) cells and may be able to attenuate allergic airway inflammation [129]. Notably, T_h_2 cells are thought to be drivers of the pathology seen in allergic asthma. Taking advantage of immune cell-derived EVs has also been applied to the treatment of ALI. One study showed that neutrophil membranes can be disrupted by nitrogen cavitation to produce EV-like particles, which can be loaded with drugs and administered to mice with ALI to attenuate inflammation [130]. Although these studies are promising, several barriers exist to translating EV-based therapies to the clinic. EVs, even those secreted by a single cell type in vitro, are heterogeneous. Improved methods of collecting, isolating, and characterizing EVs may make translation more feasible in the future.

### EVs in the clinic

Outside of the pathologies discussed in this review, EV-based therapeutics for clinical wound healing applications have recently been explored. Particularly, mesenchymal stem cell-derived EV therapies are quickly moving toward clinical applications [131]. Stem cell-based EV therapies have been evaluated in the context of post-surgical wound healing, specifically for the treatment of colo-cutaneuous fistulas and ischemia–reperfusion injury [132, 133]. Furthermore, pharmacological inhibition of EV biogenesis (i.e. through GW4869) has been proposed as a therapeutic route [134]. However, not only do malignant processes rely on EV-mediated communication, but EVs are also crucial for maintaining tissue homeostasis and carrying out routine physiological processes [135]. These inhibitors would non-specifically block the activity of EVs, which would likely lead to many off-target effects. One solution that is currently undergoing an Early Feasibility Phase I clinical trial is the depletion of patient circulating EVs (ClinicalTrials.gov: NCT04453046) [136]. An alternative method is to block the uptake of EVs in the target cell population [137, 138]. While this strategy avoids disrupting necessary communication, the approach is significantly more complex as rigorous studies need to be conducted to establish target cells and their methods of vesicular uptake. It should be noted that these EVs are likely not limited to one target organ or cell population. Indeed, the studies reviewed here have demonstrated that autocrine communication through EVs is integral to disease progression. The intricacies associated with EV-mediated communication must be considered as we look for ways to develop and improve therapies.

## Conclusions

The work discussed in this review underscores the importance of EVs in intercellular communication networks that govern tissue injury and disease progression. In the diseases and conditions explored here, healthy cells are exposed to stress- and damage-inducing conditions upon progression of the disease state. These conditions amplify EV secretion, where the ensuing EV-mediated communication results in ongoing crosstalk between immune cells and stromal and parenchymal cells. Ultimately, these interactions can reshape the tissue microenvironment to promote the survival, proliferation, and resistance of dysfunctional cells. While our understanding of EV-mediated communication is expanding rapidly, there are clear gaps in our knowledge of the role of EVs in the progression of pathological conditions. As such, much of the research highlighted here requires comprehensive follow-up studies to further solidify therapeutic targets. Nonetheless, the insights gained from these studies provide a framework to establish alternative and improved therapies for patients suffering from complex diseases.

## Data Availability

Not applicable.

## Abbreviations

ɑSMA: Alpha smooth muscle actin
ABC: ATP-binding cassette
ALI: Acute lung injury
AML: Acute myeloid leukemia
APC: Antigen-presenting cell
ARDS: Acute respiratory distress syndrome
ATM: Adipose tissue macrophages
ATP: Adenosine triphosphate
BALF: Bronchoalveolar lavage fluid
CCL: Chemokine ligand
COPD: Chronic obstructive pulmonary disease
CRC: Colorectal cancer
CSF: Colony-stimulating factor
CTC: Circulating tumor cell
ECM: Extracellular matrix
EV: Extracellular vesicle
HMGCR: 3-Hydroxy-3-methyl-glutaryl-coenzyme A reductase
HSC: Hepatic stellate cell
IL: Interleukin
IR: Ionizing radiation
KLF: Krüppel-like factor
LPS: Lipopolysaccharide
mRNA: Messenger RNA
miRNA: Micro RNA
MIF: Macrophage inhibitory factor
MLK: Mixed lineage kinase
MMP: Matrix metalloproteinase
MV: Microvesicle
NAFLD: Non-alcoholic fatty liver disease
NASH: Non-alcoholic steatohepatitis
NET: Neutrophil extracellular trap
NK: Natural killer
PA: Palmitic acid
PD-1: Programmed cell death protein 1
PD-L1: Programmed-death ligand 1
PTEN: Phosphatase and tensin homolog
RIBE: Radiation-induced bystander effect(s)
ROS: Reactive oxygen species
SIRS: Systemic inflammatory response syndrome
SOCS: Suppressor of cytokine signaling
TAM: Tumor-associated macrophage
TCA: Tricarboxylic acid
TCR: T cell receptor
TF: Tissue-factor
TGF: Transforming growth factor
TLR: Toll-like receptor
TME: Tumor microenvironment
TNF: Tumor necrosis factor
TSAP: Tumor suppressor-activated pathway
VEGF: Vascular endothelial growth factor

## References

[1] Tkach M, Théry C (2016). Communication by extracellular vesicles: where we are and where we need to go. Cell.

[2] Colombo M, Raposo G, Théry C (2014). Biogenesis, secretion, and intercellular interactions of exosomes and other extracellular vesicles. Annu Rev Cell Dev Biol.

[3] Williams C, Palviainen M, Reichardt NC, Siljander PRM, Falcón-Pérez JM (2019). Metabolomics applied to the study of extracellular vesicles. Metabolites.

[4] Hazan-Halevy I, Rosenblum D, Weinstein S, Bairey O, Raanani P, Peer D (2015). Cell-specific uptake of mantle cell lymphoma-derived exosomes by malignant and non-malignant B-lymphocytes. Cancer Lett.

[5] Toda Y, Takata K, Nakagawa Y, Kawakami H, Fujioka S, Kobayashi K (2015). Effective internalization of U251-MG-secreted exosomes into cancer cells and characterization of their lipid components. Biochem Biophys Res Commun.

[6] Rana S, Yue S, Stadel D, Zöller M (2012). Toward tailored exosomes: The exosomal tetraspanin web contributes to target cell selection. Int J Biochem Cell Biol.

[7] Fu S, Zhang Y, Li Y, Luo L, Zhao Y, Yao Y (2020). Extracellular vesicles in cardiovascular diseases. Cell Death Discov.

[8] Karpman D, Ståhl A, Arvidsson I (2017). Extracellular vesicles in renal disease. Nat Rev Nephrol.

[9] Murphy C, Withrow J, Hunter M, Liu Y, Tang YL, Fulzele S (2018). Emerging role of extracellular vesicles in musculoskeletal diseases. Mol Asp Med.

[10] Roser M, Ritchie H. Burden of Disease. Our World in Data. 2016 [cited 2021 Apr 29].

[11] Saltiel AR, Olefsky JM (2017). Inflammatory mechanisms linking obesity and metabolic disease. J Clin Investig.

[12] Gonzalez H, Hagerling C, Werb Z (2018). Roles of the immune system in cancer: from tumor initiation to metastatic progression. Genes Dev.

[13] Marsland BJ, Königshoff M, Saglani S, Eickelberg O (2011). Immune system dysregulation in chronic lung disease. Eur Respir J.

[14] Hanahan D, Weinberg RA (2011). Hallmarks of cancer: the next generation. Cell.

[15] Cooks T, Pateras IS, Jenkins LM, Patel KM, Robles AI, Morris J (2018). Mutant p53 cancers reprogram macrophages to tumor supporting macrophages via exosomal miR-1246. Nat Commun.

[16] Van Overmeire E, Laoui D, Keirsse J, Van Ginderachter JA, Sarukhan A (2014). Mechanisms driving macrophage diversity and specialization in distinct tumor microenvironments and parallelisms with other tissues. Front Immunol.

[17] Bingle L, Brown NJ, Lewis CE (2002). The role of tumour-associated macrophages in tumour progression: implications for new anticancer therapies. J Pathol.

[18] Yao Y, Xu XH, Jin L (2019). Macrophage polarization in physiological and pathological pregnancy. Front Immunol.

[19] Murray PJ (2017). Macrophage polarization. Annu Rev Physiol.

[20] Cao L, Che X, Qiu X, Li Z, Yang B, Wang S (2019). M2 macrophage infiltration into tumor islets leads to poor prognosis in non-small-cell lung cancer. Cancer Manag Res.

[21] Kurahara H, Shinchi H, Mataki Y, Maemura K, Noma H, Kubo F (2011). Significance of M2-polarized tumor-associated macrophage in pancreatic cancer. J Surg Res.

[22] Kim MJ, Sun HJ, Song YS, Yoo SK, Kim YA, Seo JS (2019). CXCL16 positively correlated with M2-macrophage infiltration, enhanced angiogenesis, and poor prognosis in thyroid cancer. Sci Rep.

[23] Zhao S, Mi Y, Guan B, Zheng B, Wei P, Gu Y (2020). Tumor-derived exosomal miR-934 induces macrophage M2 polarization to promote liver metastasis of colorectal cancer. J Hematol Oncol.

[24] Milani M, Harris AL (2008). Targeting tumour hypoxia in breast cancer. Eur J Cancer.

[25] Pouysségur J, Dayan F, Mazure NM (2006). Hypoxia signalling in cancer and approaches to enforce tumour regression. Nature.

[26] Park JE, Dutta B, Tse SW, Gupta N, Tan CF, Low JK (2019). Hypoxia-induced tumor exosomes promote M2-like macrophage polarization of infiltrating myeloid cells and microRNA-mediated metabolic shift. Oncogene.

[27] Zheng P, Luo Q, Wang W, Li J, Wang T, Wang P (2018). Tumor-associated macrophages-derived exosomes promote the migration of gastric cancer cells by transfer of functional Apolipoprotein e. Cell Death Dis.

[28] Menck K, Bleckmann A, Wachter A, Hennies B, Ries L, Schulz M (2017). Characterisation of tumour-derived microvesicles in cancer patients’ blood and correlation with clinical outcome. J Extracell Vesicles.

[29] Kaler P, Augenlicht L, Klampfer L (2009). Macrophage-derived IL-1Β stimulates Wnt signaling and growth of colon cancer cells: a crosstalk interrupted by vitamin D"3. Oncogene.

[30] Lopez-Castejon G, Brough D (2011). Understanding the mechanism of IL-1β secretion. Cytokine Growth Factor Rev.

[31] Zhao C, Bu X, Wang W, Ma T, Ma H (2014). GEC-derived SFRP5 inhibits Wnt5a-induced macrophage chemotaxis and activation. PLoS ONE.

[32] Gross JC, Chaudhary V, Bartscherer K, Boutros M (2012). Active Wnt proteins are secreted on exosomes. Nat Cell Biol.

[33] Freeman GJ, Long AJ, Iwai Y, Bourque K, Chernova T, Nishimura H (2000). Engagement of the PD-1 immunoinhibitory receptor by a novel B7 family member leads to negative regulation of lymphocyte activation. J Exp Med.

[34] Topalian SL, Drake CG, Pardoll DM (2015). Immune checkpoint blockade: a common denominator approach to cancer therapy. Cancer Cell.

[35] Haderk F, Schulz R, Iskar M, Cid LL, Worst T, Willmund KV (2017). Tumor-derived exosomes modulate PD-L1 expression in monocytes. Sci Immunol.

[36] Chen G, Huang AC, Zhang W, Zhang G, Wu M, Xu W (2018). Exosomal PD-L1 contributes to immunosuppression and is associated with anti-PD-1 response. Nature.

[37] Yang Y, Li CW, Chan LC, Wei Y, Hsu JM, Xia W (2018). Exosomal PD-L1 harbors active defense function to suppress t cell killing of breast cancer cells and promote tumor growth. Cell Res.

[38] Poggio M, Hu T, Pai CC, Chu B, Belair CD, Chang A (2019). Suppression of exosomal PD-L1 induces systemic anti-tumor immunity and memory. Cell.

[39] American Cancer Society. Cancer Treatment & Survivorship Facts & Figures 2019–2021. Am Cancer Soc. 2019.

[40] Chen Y, Jungsuwadee P, Vore M, Butterfield DA, St. Clair DK (2007). Collateral damage in cancer chemotherapy: oxidative stress in nontargeted tissues. Mol Interv.

[41] Yang H, Villani RM, Wang H, Simpson MJ, Roberts MS, Tang M (2018). The role of cellular reactive oxygen species in cancer chemotherapy. J Exp Clin Cancer Res.

[42] Yarana C, St. Clair DK (2017). Chemotherapy-induced tissue injury: An insight into the role of extracellular vesicles-mediated oxidative stress responses. Antioxidants.

[43] Carver KA, Yang D (2016). N-acetylcysteine amide protects against oxidative stress–induced microparticle release from human retinal pigment epithelial cells. Investig Ophthalmol Vis Sci.

[44] Novelli F, Neri T, Tavanti L, Armani C, Noce C, Falaschi F (2014). Procoagulant, tissue factor-bearing microparticles in bronchoalveolar lavage of interstitial lung disease patients: an observational study. PLoS ONE.

[45] Thom SR, Bhopale VM, Hu JP, Yang M (2017). Increased carbon dioxide levels stimulate neutrophils to produce microparticles and activate the nucleotide-binding domain-like receptor 3 inflammasome. Free Radic Biol Med.

[46] Shen M, Dong C, Ruan X, Yan W, Cao M, Pizzo D (2019). Chemotherapy-induced extracellular vesicle miRNAs promote breast cancer stemness by targeting OneCUT2. Cancer Res.

[47] Bai X, Ni J, Beretov J, Graham P, Li Y (2018). Cancer stem cell in breast cancer therapeutic resistance. Cancer Treat Rev.

[48] Hong CS, Jeong E, Boyiadzis M, Whiteside TL (2020). Increased small extracellular vesicle secretion after chemotherapy via upregulation of cholesterol metabolism in acute myeloid leukaemia. J Extracell Vesicles.

[49] Assmann N, O’brien KL, Donnelly RP, Dyck L, Zaiatz-Bittencourt V, Loftus RM (2017). Srebp-controlled glucose metabolism is essential for NK cell functional responses. Nat Immunol.

[50] Li S, Yan W, Yang X, Chen L, Fan L, Liu H (2019). Less micrometastatic risk related to circulating tumor cells after endoscopic breast cancer surgery compared to open surgery. BMC Cancer.

[51] Fu Q, Zhang Q, Lou Y, Yang J, Nie G, Chen Q (2018). Primary tumor-derived exosomes facilitate metastasis by regulating adhesion of circulating tumor cells via SMAD3 in liver cancer. Oncogene.

[52] Costa-Silva B, Aiello NM, Ocean AJ, Singh S, Zhang H, Thakur BK (2015). Pancreatic cancer exosomes initiate pre-metastatic niche formation in the liver. Nat Cell Biol.

[53] Chen XW, Yu TJ, Zhang J, Li Y, Chen HL, Yang GF (2017). CYP4A in tumor-associated macrophages promotes pre-metastatic niche formation and metastasis. Oncogene.

[54] Rafat M, Aguilera TA, Vilalta M, Bronsart LL, Soto LA, Von Eyben R (2018). Macrophages promote circulating tumor cell-mediated local recurrence following radiotherapy in immunosuppressed patients. Cancer Res.

[55] Zeng Z, Li Y, Pan Y, Lan X, Song F, Sun J (2018). Cancer-derived exosomal miR-25-3p promotes pre-metastatic niche formation by inducing vascular permeability and angiogenesis. Nat Commun.

[56] Aosasa S, Ono S, Mochizuki H, Tsujimoto H, Osada S-I, Takayama E (2000). Activation of monocytes and endothelial cells depends on the severity of surgical stress. World J Surg.

[57] Takahata R, Ono S, Tsujimoto H, Hiraki S, Kimura A, Kinoshita M (2011). Postoperative serum concentrations of high mobility group box chromosomal protein-1 correlates to the duration of SIRS and pulmonary dysfunction following gastrointestinal surgery. J Surg Res.

[58] Iwasaki A, Shirakusa T, Maekawa T, Enatsu S, Maekawa S (2005). Clinical evaluation of systemic inflammatory response syndrome (SIRS) in advanced lung cancer (T3 and T4) with surgical resection. Eur J Cardio-Thoracic Surg.

[59] Tohme S, Yazdani HO, Al-Khafaji AB, Chidi AP, Loughran P, Mowen K (2016). Neutrophil extracellular traps promote the development and progression of liver metastases after surgical stress. Cancer Res.

[60] Hidalgo A, Chilvers ER, Summers C, Koenderman L (2019). The neutrophil life cycle. Trends Immunol.

[61] Kobayashi SD, Voyich JM, Burlak C, DeLeo FR (2005). Neutrophils in the innate immune response. Arch Immunol Ther Exp.

[62] Brinkmann V, Reichard U, Goosmann C, Fauler B, Uhlemann Y, Weiss DS (2004). Neutrophil extracellular traps kill bacteria. Science (80-).

[63] Park J, Wysocki RW, Amoozgar Z, Maiorino L, Fein MR, Jorns J (2016). Cancer cells induce metastasis-supporting neutrophil extracellular DNA traps. Sci Transl Med..

[64] Leal AC, Mizurini DM, Gomes T, Rochael NC, Saraiva EM, Dias MS (2017). Tumor-derived exosomes induce the formation of neutrophil extracellular traps: implications for the establishment of cancer-associated thrombosis. Sci Rep.

[65] Stakos DA, Kambas K, Konstantinidis T, Mitroulis I, Apostolidou E, Arelaki S (2015). Expression of functional tissue factor by neutrophil extracellular traps in culprit artery of acute myocardial infarction. Eur Heart J.

[66] Thomas GM, Brill A, Mezouar S, Crescence L, Gallant M, Dubois C (2015). Tissue factor expressed by circulating cancer cell-derived microparticles drastically increases the incidence of deep vein thrombosis in mice. J Thromb Haemost.

[67] De Ruysscher D, Niedermann G, Burnet NG, Siva S, Lee AWM, Hegi-Johnson F (2019). Radiotherapy toxicity. Nat Rev Dis Prim.

[68] Nagasawa H, Little JB (1992). Induction of sister chromatid exchanges by extremely low doses of α-particles. Cancer Res.

[69] Seymour CB, Mothersill C (2004). Radiation-induced bystander effects—implications for cancer. Nat Rev Cancer.

[70] Yu X, Harris SL, Levine AJ (2006). The regulation of exosome secretion: a novel function of the p53 protein. Cancer Res.

[71] Lespagnol A, Duflaut D, Beekman C, Blanc L, Fiucci G, Marine JC (2008). Exosome secretion, including the DNA damage-induced p53-dependent secretory pathway, is severely compromised in TSAP6/Steap3-null mice. Cell Death Differ.

[72] Mutschelknaus L, Peters C, Winkler K, Yentrapalli R, Heider T, Atkinson MJ (2016). Exosomes derived from squamous head and neck cancer promote cell survival after ionizing radiation. PLoS ONE.

[73] Al-Mayah A, Bright S, Chapman K, Irons S, Luo P, Carter D (2015). The non-targeted effects of radiation are perpetuated by exosomes. Mutat Res - Fundam Mol Mech Mutagen.

[74] Mutschelknaus L, Azimzadeh O, Heider T, Winkler K, Vetter M, Kell R (2017). Radiation alters the cargo of exosomes released from squamous head and neck cancer cells to promote migration of recipient cells. Sci Rep.

[75] Abramowicz A, Łabaj W, Mika J, Szołtysek K, Ślęzak-Prochazka I, Mielańczyk Ł (2020). MicroRNA profile of exosomes and parental cells is differently affected by ionizing radiation. Radiat Res.

[76] Jung KO, Jo H, Yu JH, Gambhir SS, Pratx G (2018). Development and MPI tracking of novel hypoxia-targeted theranostic exosomes. Biomaterials.

[77] Mo F, Xu Y, Zhang J, Zhu L, Wang C, Chu X (2020). Effects of hypoxia and radiation-induced exosomes on migration of lung cancer cells and angiogenesis of umbilical vein endothelial cells. Radiat Res.

[78] Saklayen MG (2018). The global epidemic of the metabolic syndrome. Curr Hypertens Rep.

[79] Hales CM, Carroll MD, Fryar CD, Ogden CL (2020). Prevalence of obesity and severe obesity among adults: United States, 2017–2018. NCHS Data Brief.

[80] World Health Organization (2015). Controlling the global obesity epidemic.

[81] Martínez MC, Andriantsitohaina R (2017). Extracellular vesicles in metabolic syndrome. Circ Res.

[82] Li F, Li H, Jin X, Zhang Y, Kang X, Zhang Z (2019). Adipose-specific knockdown of Sirt1 results in obesity and insulin resistance by promoting exosomes release. Cell Cycle.

[83] Thomou T, Mori MA, Dreyfuss JM, Konishi M, Sakaguchi M, Wolfrum C (2017). Adipose-derived circulating miRNAs regulate gene expression in other tissues. Nature.

[84] Pan Y, Hui X, Chong Hoo RL, Ye D, Cheung Chan CY, Feng T (2019). Adipocyte-secreted exosomal microRNA-34a inhibits M2 macrophage polarization to promote obesity-induced adipose inflammation. J Clin Investig.

[85] Ying W, Riopel M, Bandyopadhyay G, Dong Y, Birmingham A, Seo JB (2017). Adipose tissue macrophage-derived exosomal miRNAs can modulate in vivo and in vitro insulin sensitivity. Cell.

[86] Freeman DW, Noren Hooten N, Eitan E, Green J, Mode NA, Bodogai M (2018). Altered extracellular vesicle concentration, cargo, and function in diabetes. Diabetes.

[87] Angulo MA, Royo F, Falcón-Pérez JM (2019). Metabolic nano-machines: extracellular vesicles containing active enzymes and their contribution to liver diseases. Curr Pathobiol Rep.

[88] Royo F, Palomo L, Mleczko J, Gonzalez E, Alonso C, Martínez I (2017). Metabolically active extracellular vesicles released from hepatocytes under drug-induced liver-damaging conditions modify serum metabolome and might affect different pathophysiological processes. Eur J Pharm Sci.

[89] Holman NS, Mosedale M, Wolf KK, LeCluyse EL, Watkins PB (2016). Subtoxic alterations in hepatocyte-derived exosomes: an early step in drug-induced liver injury?. Toxicol Sci.

[90] Antoine DJ, Dear JW, Lewis PS, Platt V, Coyle J, Masson M (2013). Mechanistic biomarkers provide early and sensitive detection of acetaminophen-induced acute liver injury at first presentation to hospital. Hepatology.

[91] Momen-Heravi F, Bala S, Kodys K, Szabo G (2015). Exosomes derived from alcohol-treated hepatocytes horizontally transfer liver specific miRNA-122 and sensitize monocytes to LPS. Sci Rep.

[92] Ju L, Sun Y, Xue H, Chen L, Gu C, Shao J (2020). CCN1 promotes hepatic steatosis and inflammation in non-alcoholic steatohepatitis. Sci Rep.

[93] Ibrahim SH, Hirsova P, Tomita K, Bronk SF, Werneburg NW, Harrison SA (2016). Mixed lineage kinase 3 mediates release of C-X-C motif ligand 10-bearing chemotactic extracellular vesicles from lipotoxic hepatocytes. Hepatology.

[94] Tomita K, Freeman BL, Bronk SF, LeBrasseur NK, White TA, Hirsova P (2016). CXCL10-mediates macrophage, but not other innate immune cells-associated inflammation in murine nonalcoholic steatohepatitis. Sci Rep.

[95] Younossi ZM, Koenig AB, Abdelatif D, Fazel Y, Henry L, Wymer M (2016). Global epidemiology of nonalcoholic fatty liver disease—meta-analytic assessment of prevalence, incidence, and outcomes. Hepatology.

[96] Lee YS, Kim SY, Ko E, Lee JH, Yi HS, Yoo YJ (2017). Exosomes derived from palmitic acid-treated hepatocytes induce fibrotic activation of hepatic stellate cells. Sci Rep.

[97] Hirsova P, Gores GJ (2015). Death receptor-mediated cell death and proinflammatory signaling in nonalcoholic steatohepatitis. CMGH.

[98] Admyre C, Grunewald J, Thyberg J, Bripenäck S, Tornling G, Eklund A (2003). Exosomes with major histocompatibility complex class II and co-stimulatory molecules are present in human BAL fluid. Eur Respir J.

[99] Pua HH, Happ HC, Gray CJ, Mar DJ, Chiou NT, Hesse LE (2019). Increased hematopoietic extracellular RNAs and vesicles in the lung during allergic airway responses. Cell Rep.

[100] Eltom S, Dale N, Raemdonck KRG, Stevenson CS, Snelgrove RJ, Sacitharan PK (2014). Respiratory infections cause the release of extracellular vesicles: implications in exacerbation of asthma/COPD. PLoS ONE.

[101] Hough KP, Wilson LS, Trevor JL, Strenkowski JG, Maina N, Kim YI (2018). Unique lipid signatures of extracellular vesicles from the airways of asthmatics. Sci Rep.

[102] Soni S, Wilson MR, O’Dea KP, Yoshida M, Katbeh U, Woods SJ (2016). Alveolar macrophage-derived microvesicles mediate acute lung injury. Thorax.

[103] Ye C, Li H, Bao M, Zhuo R, Jiang G, Wang W (2020). Alveolar macrophage-derived exosomes modulate severity and outcome of acute lung injury. Aging (Albany NY).

[104] MacKenzie A, Wilson HL, Kiss-Toth E, Dower SK, North RA, Surprenant A (2001). Rapid secretion of interleukin-1β by microvesicle shedding. Immunity.

[105] Shelke GV, Yin Y, Jang SC, Lässer C, Wennmalm S, Hoffmann HJ (2019). Endosomal signalling via exosome surface TGFβ-1. J Extracell Vesicles.

[106] Fitzgerald W, Freeman ML, Lederman MM, Vasilieva E, Romero R, Margolis L (2018). A system of cytokines encapsulated in extracellular vesicles. Sci Rep.

[107] Majumdar R, Tavakoli Tameh A, Parent CA (2016). Exosomes mediate LTB4 release during neutrophil chemotaxis. PLoS Biol.

[108] Esser J, Gehrmann U, D’Alexandri FL, Hidalgo-Estévez AM, Wheelock CE, Scheynius A (2010). Exosomes from human macrophages and dendritic cells contain enzymes for leukotriene biosynthesis and promote granulocyte migration. J Allergy Clin Immunol.

[109] Torregrosa Paredes P, Esser J, Admyre C, Nord M, Rahman QK, Lukic A (2012). Bronchoalveolar lavage fluid exosomes contribute to cytokine and leukotriene production in allergic asthma. Allergy Eur J Allergy Clin Immunol.

[110] Blanchard N, Lankar D, Faure F, Regnault A, Dumont C, Raposo G (2002). TCR activation of human T cells induces the production of exosomes bearing the TCR/CD3/ζ complex. J Immunol.

[111] Genschmer KR, Russell DW, Lal C, Szul T, Bratcher PE, Noerager BD (2019). Activated PMN exosomes: pathogenic entities causing matrix destruction and disease in the lung. Cell.

[112] Li CJ, Liu Y, Chen Y, Yu D, Williams KJ, Liu ML (2013). Novel proteolytic microvesicles released from human macrophages after exposure to tobacco smoke. Am J Pathol.

[113] Szul T, Bratcher PE, Fraser KB, Kong M, Tirouvanziam R, Ingersoll S (2016). Toll-like receptor 4 engagement mediates prolyl endopeptidase release from airway epithelia via exosomes. Am J Respir Cell Mol Biol.

[114] Moon HG, Cao Y, Yang J, Lee JH, Choi HS, Jin Y (2015). Lung epithelial cell-derived extracellular vesicles activate macrophage-mediated inflammatory responses via ROCK1 pathway. Cell Death Dis.

[115] Lee H, Zhang D, Wu J, Otterbein LE, Jin Y (2017). Lung epithelial cell-derived microvesicles regulate macrophage migration via MicroRNA-17/221-induced integrin β 1 recycling. J Immunol.

[116] Lee H, Zhang D, Zhu Z, Dela Cruz CS, Jin Y (2016). Epithelial cell-derived microvesicles activate macrophages and promote inflammation via microvesicle-containing microRNAs. Sci Rep.

[117] Shikano S, Gon Y, Maruoka S, Shimizu T, Kozu Y, Iida Y (2019). Increased extracellular vesicle miRNA-466 family in the bronchoalveolar lavage fluid as a precipitating factor of ARDS. BMC Pulm Med.

[118] Cañas JA, Sastre B, Rodrigo-Muñoz JM, Fernández-Nieto M, Barranco P, Quirce S (2018). Eosinophil-derived exosomes contribute to asthma remodelling by activating structural lung cells. Clin Exp Allergy.

[119] Lee H, Zhang D, Laskin DL, Jin Y. Functional Evidence of Pulmonary Extracellular Vesicles in Infectious and Noninfectious Lung Inflammation. J Immunol. 2018; 10.4049/jimmunol.1800264 PMC6109965 29997122

[120] Bourdonnay E, Zasłona Z, Penke LRK, Speth JM, Schneider DJ, Przybranowski S (2015). Transcellular delivery of vesicular SOCS proteins from macrophages to epithelial cells blunts inflammatory signaling. J Exp Med.

[121] Draijer C, Speth JM, Penke LRK, Zaslona Z, Bazzill JD, Lugogo N (2020). Resident alveolar macrophage-derived vesicular SOCS3 dampens allergic airway inflammation. FASEB J.

[122] Wang J, Cai Zhang Z, Lin Z, Fei X, Zhang F, Shuangshuang Wan F (2018). CD8α+ CD11c+ extracellular vesicles in the lungs control immune homeostasis of the respiratory tract via TGF-β1 and IL-10. J Immunol.

[123] Yao MY, Zhang WH, Ma WT, Liu QH, Xing LH, Zhao GF (2019). microRNA-328 in exosomes derived from M2 macrophages exerts a promotive effect on the progression of pulmonary fibrosis via FAM13A in a rat model. Exp Mol Med.

[124] Levänen B, Bhakta NR, Torregrosa Paredes P, Barbeau R, Hiltbrunner S, Pollack JL (2013). Altered microRNA profiles in bronchoalveolar lavage fluid exosomes in asthmatic patients. J Allergy Clin Immunol.

[125] Bartel S, La Grutta S, Cilluffo G, Perconti G, Bongiovanni A, Giallongo A (2020). Human airway epithelial extracellular vesicle miRNA signature is altered upon asthma development. Allergy.

[126] Mayer L, Sandborn WJ, Stepanov Y, Geboes K, Hardi R, Yellin M (2014). Anti-IP-10 antibody (BMS-936557) for ulcerative colitis: A phase II randomised study. Gut.

[127] Yellin M, Paliienko I, Balanescu A, Ter-Vartanian S, Tseluyko V, Xu L-A (2012). A phase II, randomized, double-blind, placebo-controlled study evaluating the efficacy and safety of MDX-1100, a fully human anti-CXCL10 monoclonal antibody, in combination with methotrexate in patients with rheumatoid arthritis. Arthritis Rheum.

[128] Andrews DW, Judy KD, Scott CB, Garcia S, Harshyne LA, Kenyon L (2021). Phase Ib clinical trial of IGV-001 for patients with newly diagnosed glioblastoma. Clin Cancer Res.

[129] Zhang HP, Sun YX, Lin Z, Yang G, Liu JQ, Mo LH (2020). CARsomes inhibit airway allergic inflammation in mice by inducing antigen-specific Th2 cell apoptosis. Allergy Eur J Allergy Clin Immunol.

[130] Gao J, Wang S, Wang Z (2017). High yield, scalable and remotely drug-loaded neutrophil-derived extracellular vesicles (EVs) for anti-inflammation therapy. Biomaterials.

[131] Witwer KW, Van Balkom BWM, Bruno S, Choo A, Dominici M, Gimona M (2019). Defining mesenchymal stromal cell (MSC)-derived small extracellular vesicles for therapeutic applications. J Extracell Vesicles.

[132] Berger A, Araújo-Filho I, Piffoux M, Nicolás-Boluda A, Grangier A, Boucenna I (2021). Local administration of stem cell-derived extracellular vesicles in a thermoresponsive hydrogel promotes a pro-healing effect in a rat model of colo-cutaneous post-surgical fistula. Nanoscale.

[133] Ali M, Pham A, Wang X, Wolfram J, Pham S (2020). Extracellular vesicles for treatment of solid organ ischemia–reperfusion injury. Am J Transplant.

[134] Trajkovic K, Hsu C, Chiantia S, Rajendran L, Wenzel D, Wieland F (2008). Ceramide triggers budding of exosome vesicles into multivesicular endosomes. Science (80-).

[135] Luzio JP, Hackmann Y, Dieckmann NMG, Griffiths GM (2014). The Biogenesis of lysosomes and lysosome-related organelles. Cold Spring Harb Perspect Biol.

[136] Marar C, Starich B, Wirtz D (2021). Extracellular vesicles in immunomodulation and tumor progression. Nat Immunol.

[137] Mathieu M, Martin-Jaular L, Lavieu G, Théry C (2019). Specificities of secretion and uptake of exosomes and other extracellular vesicles for cell-to-cell communication. Nat Cell Biol.

[138] Mulcahy LA, Pink RC, Carter DRF (2014). Routes and mechanisms of extracellular vesicle uptake. J Extracell Vesicles.

